# Time of exposure to social defeat stress during childhood and adolescence and redox dysregulation on long-lasting behavioral changes, a translational study

**DOI:** 10.1038/s41398-022-02183-7

**Published:** 2022-09-26

**Authors:** Mirko Schnider, Raoul Jenni, Julie Ramain, Sara Camporesi, Philippe Golay, Luis Alameda, Philippe Conus, Kim Q. Do, Pascal Steullet

**Affiliations:** 1 Center for Psychiatric Neuroscience, Department of Psychiatry, Lausanne University Hospital (CHUV), 1008 Prilly-Lausanne, Switzerland; 2 Service of General Psychiatry, Department of Psychiatry, Lausanne University Hospital (CHUV), 1008 Prilly-Lausanne, Switzerland

**Keywords:** Neuroscience, Schizophrenia

## Abstract

Traumatic events during childhood/early adolescence can cause long-lasting physiological and behavioral changes with increasing risk for psychiatric conditions including psychosis. Genetic factors and trauma (and their type, degree of repetition, time of occurrence) are believed to influence how traumatic experiences affect an individual. Here, we compared long-lasting behavioral effects of repeated social defeat stress (SD) applied during either peripuberty or late adolescence in adult male WT and Gclm-KO mice, a model of redox dysregulation relevant to schizophrenia. As SD disrupts redox homeostasis and causes oxidative stress, we hypothesized that KO mice would be particularly vulnerable to such stress. We first found that peripubertal and late adolescent SD led to different behavioral outcomes. Peripubertal SD induced anxiety-like behavior in anxiogenic environments, potentiated startle reflex, and increased sensitivity to the NMDA-receptor antagonist, MK-801. In contrast, late adolescent SD led to increased exploration in novel environments. Second, the long-lasting impact of peripubertal but not late adolescent SD differed in KO and WT mice. Peripubertal SD increased anxiety-like behavior in anxiogenic environments and MK-801-sensitivity mostly in KO mice, while it increased startle reflex in WT mice. These suggest that a redox dysregulation during peripuberty interacts with SD to remodel the trajectory of brain maturation, but does not play a significant role during later SD. As peripubertal SD induced persisting anxiety- and fear-related behaviors in male mice, we then investigated anxiety in a cohort of 89 early psychosis male patients for whom we had information about past abuse and clinical assessment during the first year of psychosis. We found that a first exposure to physical/sexual abuse (analogous to SD) before age 12, but not after, was associated with higher anxiety at 6–12 months after psychosis onset. This supports that childhood/peripuberty is a vulnerable period during which physical/sexual abuse in males has wide and long-lasting consequences.

## Introduction

Exposure to environmental risk factors at different stages of brain development ranging from prenatal period (maternal stress, exposure to viral infection, birth complications), childhood (trauma such as emotional/physical neglect, physical/sexual abuse at their extreme forms; migration in adversity), pubertal and late adolescence (including drug abuse), have been associated to psychiatric disorders such as schizophrenia and affective psychoses [1, 2]. Childhood trauma (CT) and adversities increase the risk of psychotic disorders [3] and the likelihood to convert to psychosis in individuals with high-risk to psychosis [4]. Furthermore, patients who have been exposed to CT show more severe symptoms and worse functional outcomes than patients not reporting maltreatment [5–7]. Some forms of severe abuses with first exposure before 12 years old have the most robust association with poor functional outcome [5]. Thus, increasing evidence suggests that the time at first exposure to trauma, but also the type of traumatic/adverse experiences determine their impact on later outcome [8–11]. Moreover, studies report either additive or synergistic interactions between CT and polygenetic risk score for schizophrenia [12–14]. However, CT heightens also the risk for other psychiatric disorders (including PTSD, borderline personality disorder, anxiety, depression, and drug addiction) by impacting multiple symptom domains [15, 16]. Therefore, interactions between genes and the nature and timing of CT may define the incidence, the course, and prognosis of various psychiatric disorders [17]. However, a deeper understanding of the interplay between CT (its nature and the age at first exposure) and genetic vulnerability that drives persistent defective behavioral alterations is challenging within the complex naturalistic setting of epidemiological investigations. In this context, animal studies where genetic liability and stressors (exposure time, severity, nature) can be well-controlled provide an opportunity to interrogate some of these interactions.

Animal research shows that adolescence is a highly vulnerable period to stress [18, 19]. However, few studies have explicitly compared the impact of stress exposure during different periods of childhood and adolescence on adult behavioral phenotypes. Furthermore, most investigations in young post-weaned or adolescent rodents use chronic unpredictable stress consisting of random exposure to a variety of stressors, and therefore cannot explore the effect of the nature of stress.

In this respect, chronic social defeat (SD), which consists in briefly exposing a young mouse to an aggressive adult congener during several consecutive days, is analogous to physical/sexual abuse (PSA) in human. Indeed, the young mouse endures chasing, brief attacks, and sexual displays from the aggressor. In rodents, SD causes peripheral and brain oxidative stress, decreases antioxidant capacity [20–22], and promotes neuroinflammatory conditions via interactions between CNS and peripheral immune systems [23]. Likewise, childhood maltreatment in human has been associated with peripheral oxidative stress and altered antioxidant systems in adolescents without psychiatric disorders [24] but also in individuals with psychiatric conditions. Thus, early psychosis patients who suffered from CT and display a high blood oxidation status show severe clinical symptoms and cognitive deficits [25].

Our first aim was to assess in mice the persistent impact of chronic SD applied during either peripuberty (P30–40, also described as pubescence [26] or adolescence [27]) or late adolescence (P50–60) on several behavioral phenotypes (locomotor activity in novel environments, exploration in anxiogenic environments, social interaction, pre-pulse inhibition and startle reactivity, and MK-801-induced hyperlocomotion). We also investigated how a genetic vulnerability to redox dysregulation relevant to schizophrenia and possibly other psychiatric disorders [28] modulates the long-lasting impact of SD when applied respectively during peripuberty and late adolescence. We postulated that a susceptibility to redox dysregulation exacerbates some effects of SD, since SD causes oxidative stress. As model of redox dysregulation, we used Gclm knockout (Gclm-KO) mice that have decreased levels of glutathione (GSH) [29]. These mice are susceptible to oxidative stress and microglia activation [30, 31] and are pertinent to schizophrenia as GSH deficit is reported in anterior cingulate cortex and thalamus of some patients [32, 33]. Finally, in a translational perspective, we then explored in a cohort of early psychosis patients (EPP) the links between time of first exposure to PSA (before 12 versus between 12 and 16 years old) and seven psychopathological dimensions, with an emphasis on anxiety.

## Materials and methods

### Preclinical study

Animals. Experimental mice (Gclm-KO and WT, backcrossed with C57BL/6J mice) were generated by breeding heterozygotes Gclm mice [29], weaned and genotyped at P21, and kept in cages of 2–4 animals under a reversed 12-hour light-dark cycle with free access to food and water. Only males were used because of the nature of the SD stress relying on repeated interactions with male aggressors. Experiments were approved by the Local Veterinary Authorities and ethical committee.

**Fig. 1:**
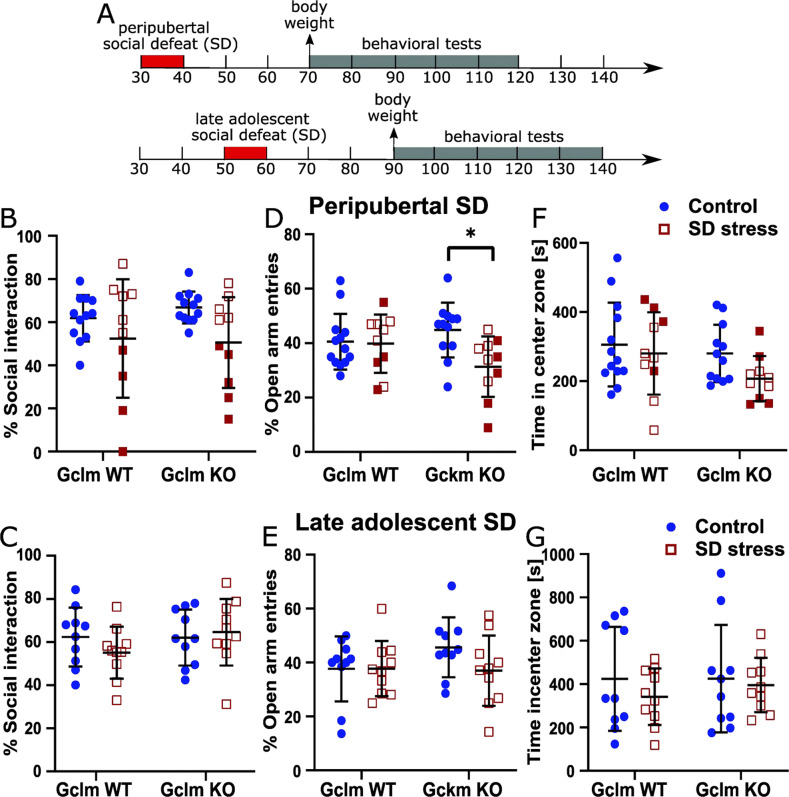
Long-lasting effects of peripubertal and late adolescent social defeat (SD) stress on social interaction and anxiety-like behavior in WT and Gclm-KO mice. **A** Schematic diagram of the experimental design. **B**, **C** Long-lasting effect of peripubertal (**B**) and late adolescent (**C**) SD on social interaction. No stress and genotype effect in both **B** and **C**. Note a subset of mice (filled squares) exposed to peripubertal SD that show strong reduction in social interactions (regardless of the genotype). **D**, **E** Long-lasting effect of peripubertal (**D**) and late adolescent (**E**) SD on open arm entries (% open arm entries relative to total entries in open and close arms) in an elevated-plus-maze (EPM). Significant peripubertal SD effect and trend for peripubertal SD × genotype interaction (in **D**). **F**, **G** Long-lasting effect of peripubertal (**F**) and late adolescent (**G**) SD on the time spend in the center zone of an open field. Control mice are non-stressed individuals. Data are presented with the mean and s.d. Post-hoc test: *p < 0.05.

SD protocol. Experimental mice were exposed to a repeated SD stress (adapted from [34]) either during peripuberty (P30 to P40) or late adolescence (P50 to P60). The stress consisted in introducing an experimental mouse into the cage of a single-housed adult male SJL mouse (from Charles River, France). After 10 minutes of physical interactions or as soon as 10 attacks from the SJL mouse occurred, a perforated Plexiglas partition was introduced to separate the two mice until the next day. This was repeated for eleven consecutive days and the experimental mice were then single-housed until behavioral testing (Fig. 1) as social grouping dampens down the persistent stress-induced behavioral alterations. Control non-stressed mice were kept grouped and gently handled during the same period of stress exposure (either P30–40 or P50–60). More details are provided in Supplemental material.

Behavioral testing. The behavior evaluation started 30 days after the end of the stress protocol (Fig. 1A). We tested 23 (13 stressed, 10 non-stressed) WT and 22 (12, 10) KO mice for the peribubertal stress experiment, and 20 (10, 10) WT and 20 (10, 10) KO mice for the late adolescent stress experiment. The behavioral tests (elevated-plus maze (EPM), open field (OF), social interaction, pre-pulse inhibition (PPI), MK-801 sensitivity) are fully described in Supplemental material, together with their order and timing (Table S1). All behavioral assays were performed during the active circadian phase.

Statistics. The body weight and each behavioral output were subjected to 2-Way ANOVAs with genotype and stress as variables followed by post-hoc multiple comparisons using the Tukey test (GraphPad Prism 8, USA). When data were not normal based on the Shapiro-Wilk test, they were log-transformed prior to perform the ANOVAs. Three-way ANOVAs were also applied with genotype and stress as independent variables, and time bins (for locomotor activity in the open field and MK-801-induced locomotion test) or prepulse intensities (for PPI) as repeated measures. When the homogeneity of variances was not met based on the Levene’s test, the Games-Howell test for multiple comparisons were used (SPSS, IBM, USA).

The changes in behavioral responses induced by stress were also compared in Gclm-WT and KO mice. Each behavioral output of a stressed mouse was normalized to the same mean output of non-stressed mice from the same genotype by computing a Z-score. For each stressed mouse, a composite anxiety Z-score was calculated by averaging the individual Z-scores for the % entry in the open arms of the EPM and for the time spent in the central zone of the OF. A composite Z-score for activity in novel environments, was computed by averaging the individual Z-scores for the total distance moved in EPM, OF, and in the arenas used during the habituation phases of the social interaction and MK-801 sensitivity tests. For each stressed mouse, a Z-score (normalized change caused by stress) was thus obtained for anxiety, activity, social interaction, startle reactivity, PPI, and MK-801-induced locomotion. A multivariate analysis (SPSS, IBM, USA) was first conducted to see whether Z-scores differed overall in Gclm-WT and KO mice. If so, for each behavioral dimension, the Z-scores of KO and WT mice were compared with a t-test or a Welch’s t-test (when unequal variances between groups) followed by a Holm Bonferroni correction for multiple comparisons. Statistical significance was set at *α* = 0.05 (two-sided).

### Clinical study

Subjects. EPP were enrolled within the Treatment and Early Intervention in Psychosis Program (TIPP, Lausanne University Hospital) [35]. Inclusion criteria comprised age between 18 and 35, having crossed the psychosis threshold according to the Comprehensive Assessment of At-Risk Mental States (CAARMS) criteria [36], no previous treatment with antipsychotic medication for more than 24 weeks, no psychosis related to intoxication or organic brain disease, intelligence quotient ≥70, and ability to provide informed consent. For a better translation with our study on male mice, we investigated only men (89) for which we had complete clinical data and information on trauma and adverse events. The local Ethics Committee (*Commission Cantonale d’Ethique de la Recherche sur l’Etre Humain*) granted access to TIPP clinical data including demographic, PANSS and trauma history. See Supplementary information, for more details.

### Psychopathological assessments

Clinical symptoms were typically evaluated at 2 and then every 6 months after entry to the TIPP by trained psychologists using the Positive And Negative Syndrome Scale (PANSS) [37]. The clinical evaluations were grouped according to the delay from psychosis threshold: <6 months and from 6 to 12 months. Based on the results of the preclinical study, anxiety was defined as a primary outcome and evaluated with the seven-factor model proposed by Emsley et al. [38] for individuals with recent-onset psychosis to categorize the PANSS items [anxiety score defined by the sum of G1 (*Somatic concern*), G2 (*Anxiety*) and G4 (*Tension*) items]. The other psychopathological dimensions identified by this model [positive and negative symptoms, disorganized (cognitive) symptoms, excited features, motor symptoms, and depression] were also explored as secondary outcomes.

Assessment of traumatic events. Clinicians were trained to conduct an extensive assessment of patients, including evaluation of exposure to traumatic life events [39]. Case managers met patients frequently over the 36-month treatment period, providing the framework to establish a trusting relationship and gather extensive knowledge of patients’ history. If patients agreed, information was completed with their family. In case of inconsistency between patient’s and family’s reports or doubt regarding the exposure to trauma or the age at the time of exposure, patients were not included in the study. Over the 3-year period of treatment, case managers completed a table containing the following information: (1) presence or absence of physical abuse, sexual abuse, emotional abuse, emotional neglect, physical neglect, migration in adversity [40], bullying, adoption, parent separation, abandon, loss of parents, (2) age at the time of first exposure to these events and (3) recurrence of trauma. Here, we focused on physical/sexual abuses (PSA) as they were quite analogous to the SD used in our animal study. Patients reporting physical and/or sexual abuses were categorized according to their first exposure to one of these abuses, respectively before the age of 12 (early PSA) and between 12 and 16 years old (late PSA). Non-trauma patients were those who did not report any of the following events (physical, sexual or emotional abuse, emotional or physical neglect, bullying, migration in adversity), even during the prodromal phase or after the onset of psychosis.

### Statistical analyses

Mixed effects models repeated measure analysis of variance (MMRM) were used to analyze differences in psychopathological measurements among the three trauma groups (no trauma, early PSA, late PSA) over the first year of psychosis (0–6 and 6–12 months). Anxiety (primary outcome) and the other psychopathological dimensions (secondary outcomes) were considered in separate MMRM models (SPSS, IBM, USA). We were interested in main, simple and interaction effects, but also in multiple comparisons (post-hoc) when a signal was present. Antipsychotic and anxiolytic medication were not used as covariate due to missing information for a significant proportion of patients. More details in Supplementary information.

## Results

### Preclinical study

The long-lasting effects of repeated SD occurring during either the peripubertal (P30–40) or late adolescent (P50–60) period on body weight and behavior were assessed in adult Gclm-WT and KO mice (Fig. 1A). We found no stress effect on weight at P70 (30 days after peripubertal stress) and P90 (30 days after late adolescent stress). However, we observed a genotype affect at P70 (*F*_1,81_ = 19.66, *P* < 0.0001) and P90 (*F*_1,36_ = 20.72, *P* < 0.0001), with lower weight in KO than in WT mice (Fig. S1).

### Social interaction

We found neither genotype nor SD effect on social interaction towards an unfamiliar C57Bl/6J mouse, regardless of the time of stress exposure (Fig. 1B, C). Following peripubertal, but not late adolescent SD, a subset of mice, irrespective of their genotype, displayed however a strong reduction in social interactions (Fig. 1B), as illustrated by the significantly larger variance of social interaction in stressed as compared to non-stressed groups (F-test, for WT: *P* = 0.005; for KO: *P* = 0.002).

### Anxiety-like behaviors

Peripubertal SD reduced the percentage of entries into the open arms of EPM (stress effect: *F*_1,41_ = 5.056, *P* = 0.03; Fig. 1D). This was mainly driven by stressed KO mice, with a close to significant genotype x stress interaction (*F*_1,41_ = 4.057, *P* = 0.0506). Thus, the percentage of entries into the open arms was significantly lower in KO mice exposed to peripubertal SD as compared to non-stressed KO mice (*P* = 0.0235, Fig. 1D). In contrast, late adolescent SD did not affect anxiety-like behavior in EPM regardless of genotype (Fig. 1E). The time spent in the center of OF was also used as another parameter to assess anxiety-like phenotype. We found no effect of stress, genotype, or interaction in both peripubertal and late adolescent SD cohorts (Fig. 1F, G). However, there was a trend that socially defeated mice during peripuberty spent less time in the central zone (Fig. 1G).

### Locomotor activity in novel environments

**Fig. 2:**
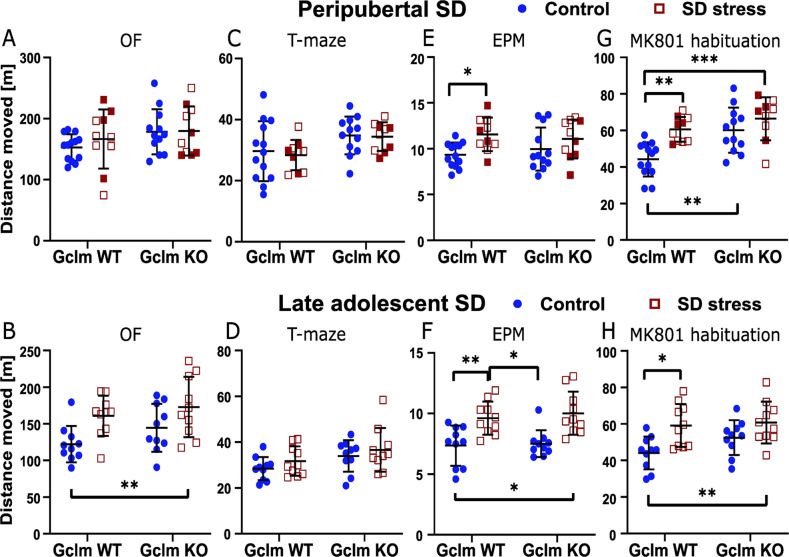
Long-lasting effects of peripubertal and late adolescent social defeat (SD) stress on exploratory activity in novel environments in WT and Gclm-KO mice. **A**, **B** open field (OF), **C**, **D** T-maze, **E**, **F** elevated-plus maze (EPM), and **G**, **H** arena used for the MK-801 test. Significant peripubertal SD effect in **E** and **G**; significant late adolescent SD effect in **B**, **F**, and **H**; significant genotype effect in **G**. Filled squares depict mice exposed to peripubertal SD that display strong reduction in social interaction. Control mice are non-stressed individuals. Data are presented with the mean and s.d. Post-hoc test: **p < 0.01, **p < 0.01, ***p < 0.001.

Overall, mice exposed to SD during late adolescence, but not prepuberty, showed an increase in locomotor activity in OF, irrespective of the genotype (late adolescent stress effect: *F*_1,36_ = 10.83, *P* = 0.0022; Figs. 2A, B and S2). We further analyzed the locomotor activity of mice in the T-maze used for the social interaction test, the EPM, and the arena used for the MK-801 test (during the habituation period). In T-maze, we observed no stress effect in both peripubertal and late adolescent SD cohorts (Fig. 2C, D). In EPM, both peripubertal and late adolescent SD affected significantly the activity (peripubertal stress: *F*_1,41_ = 8.255, *P* = 0.0064, Fig. 2E; late adolescent stress: *F*_1,36_ = 25.47, *P* < 0.0001, Fig. 2F), regardless of the genotype. Overall, stressed mice moved significantly more in EPM than their non-stressed counterparts. Finally during the habituation stage of the MK-801 test, both peripubertal and late adolescent SD increased the activity (peripubertal stress: *F*_1,40_ = 13.07, *P* = 0.0008, Fig. 2G; late adolescent stress: *F*_1,36_ = 12.2891, *P* = 0.0012, Fig. 2H). A genotype effect was also observed in the peripubertal cohort (*F*_1,40_ = 11.93, *P* = 0.0013), with KO mice being more active as compared to WT (Fig. 2G).

### Pre-pulse inhibition and startle response

**Fig. 3:**
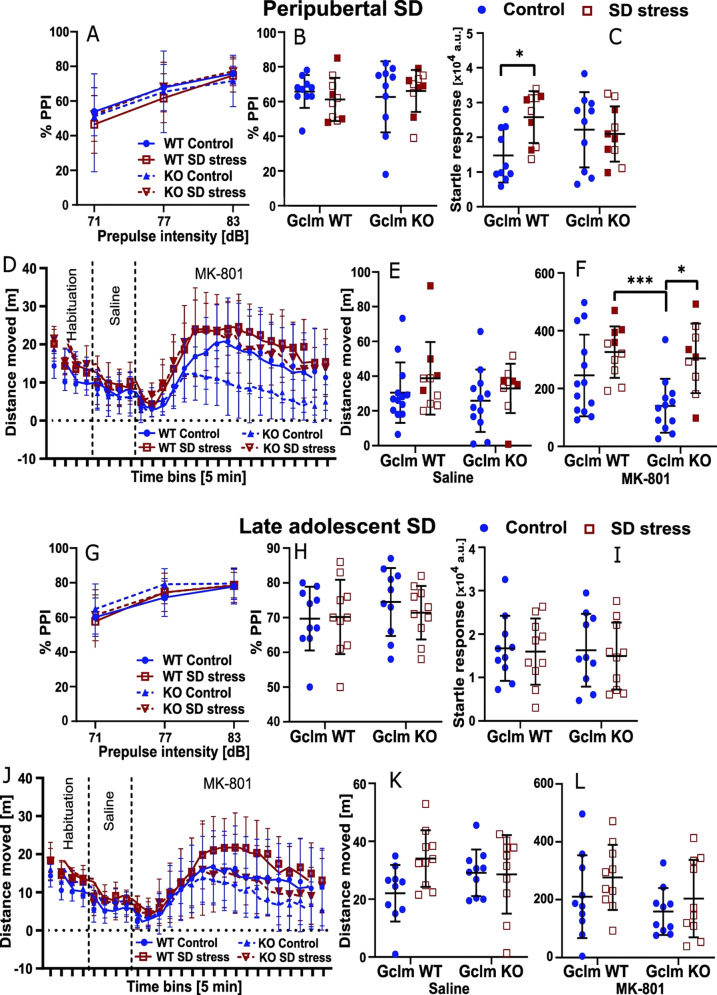
Long-lasting effects of peripubertal and late adolescent social defeat (SD) stress on acoustic prepulse inhibition (PPI), startle response, and MK-801-induced locomotor activity in WT and Gclm-KO mice. **A**, **B**, **G**, **H** Long-lasting effect of peripubertal (**A**, **B**) and late adolescent (**G**, **H**) SD on PPI. **A**, **G** PPI at three pre-pulse intensities; **B**, **H** averaged PPI considering all three pre-pulse intensities together. **C**, **I** Long-lasting effect of peripubertal (**C**) and late adolescent (**I**) SD on startle response to 100 dB sound. Significant peripubertal SD × genotype interaction in **C**, with higher response in WT exposed to peripubertal SD as compared to non-stressed WT. **D**–**F**, **J**–**L** Long-lasting effect of peripubertal (**D**–**F**) and late adolescent (**J**–**L**) SD on the behavior in the MK-801test. **D**, **J** Recorded locomotor activity along the 3 phases of the test: 20-min habituation, 20-min following saline injection, 90-min following MK-801 injection. **E**, **K** Overall locomotor activity during saline phase. **F**, **L** Overall locomotor activity during MK-801 phase. Significant peripubertal SD effect and trend for genotype effect in **F**. During the MK-801 phase, significant interactions between time bins and peripubertal SD (**D**) and between time bins and genotype (**D**, **J**). Filled squares depict mice exposed to peripubertal SD that display strong reduction in social interaction. Control mice are non-stressed individuals. Data are presented with the mean and s.d. post-hoc test: *p < 0.05, **p < 0.01.

The inhibition of the startle response to a brief loud sound when preceded by a weak pre-pulse auditory stimulus (pre-pulse inhibition, PPI) was used to evaluate sensory-motor gating. We found no effect of stress (peripubertal or late adolescent), genotype, or interaction on PPI (Fig. 3A, B, G, H). Regarding the startle response, we observed a genotype × peripubertal stress interaction (*F*_F1,36_ = 5.064, *P* = 0.0306) due to increased startle response in WT mice exposed to peripubertal SD as compared to non-stressed counterparts (*P* = 0.0333, Fig. 3C). By contrast, there was neither stress nor genotype effects, nor interaction on the startle response in the late adolescent stress cohort (Fig. 3I).

### MK-801-induced hyperlocomotion

The locomotion induced by the MK-801 was measured during respectively the habituation, saline, and MK-801 stages (Fig. 3D, J). During the saline phase, we observed no main effects or interaction (Fig. 3E, K), with low locomotor activity in all groups. Following MK-801 injection, mice exhibited increased locomotor activity, independently of the genotype and stress type (Fig. 3D, J). In the peripubertal stress cohort, we found a stress effect (*F*_1,40_ = 12.37, *P* = 0.0011) and a trend for genotype effect (*F*_1,40_ = 3.312, *P* = 0.0763) on the total distance traveled during 90 min following MK-801 administration (Fig. 3F). MK-801-induced increased locomotion was the weakest in non-stressed KO mice, particularly beyond 30–40 min post MK-801 administration (Fig. 3D, F). In the peripubertal stress cohort, a 3-way ANOVA with time bins as repeated measures showed indeed interactions between genotype and time bins (*F*_17,680_ = 4.357, *P* < 0.0001), and between stress and time bins (*F*_17,680_ = 2.273, *P* = 0.0025). Together, this indicates that KO mice were less sensitive to MK-801 and a peripubertal SD exposure strongly sensitized KO mice to MK-801. In the late adolescent stress cohort, we found no significant effect of stress, genotype, and interactions (Fig. 3J, L). However, a three-way ANOVA with time bins as repeated measures revealed an interaction between genotype and time bins (*F*_17,612_ = 4.181, *P* < 0.0001), further supporting that non-stressed KO mice were less sensitive and responded to MK-801 during a shorter period as compared to non-stressed WT mice.

### Peripuberty is more vulnerable to SD and genetic redox dysregulation

**Fig. 4:**
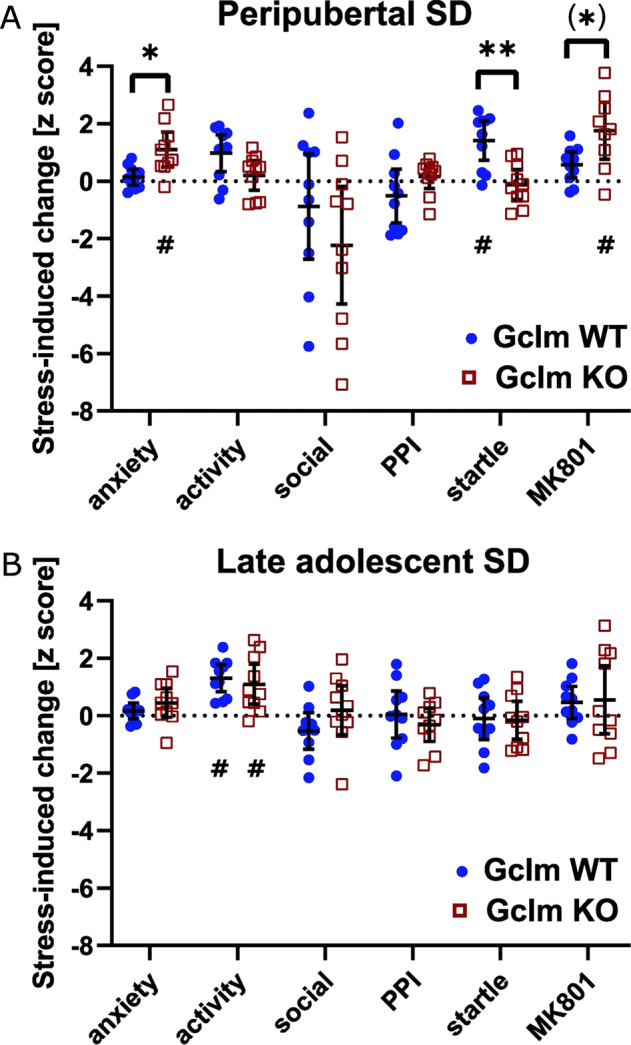
Stress-induced long-lasting changes in anxiety-like behavior in anxiogenic environment, exploratory activity in novel environments, social interaction, pre-pulse inhibition (PPI), acoustic startle reflex, and MK-801-induced locomotor activity in WT and Gclm-KO mice. The behavioral changes (*z* scores) induced by peripubertal SD stress (**A**), but not late adolescent SD stress (**B**), are significantly different in both genotypes. Data are presented with the mean and 95% confidence interval. * indicates differences between WT and KO mice: *p < 0.05, **p < 0.01, (*) did not survive Holm Bonferroni for multiple comparisons. ^#^Indicates that stress significantly affected one behavioral dimension (*p* < 0.05, sign test).

Overall, the data suggest that SD during peripuberty, but not late adolescence, impacts differently WT and Gclm-KO mice. To further explore this, we computed for each stressed mouse a *Z*-score of each behavioral output based on the data from non-stressed mice of the same genotype. This provided for each genotype a normalized measure of how each behavior of stressed mice deviated from those of non-stressed individuals. We then compared the *Z*-scores for anxiety-like behavior in anxiogenic environments, exploratory activity in novel environments, social interaction, startle reactivity, PPI, and MK-801-induced locomotion of stressed Gclm-KO and WT mice using a multivariate analysis. In the prepubertal stress but not late adolescence stress cohort, we found an overall significant difference between both genotypes (*P* = 0.013), indicating that peripubertal SD affected differently KO and WT mice. A subsequent post-hoc analysis revealed differences between genotypes for anxiety (*P* = 0.005), startle reactivity (*P* = 0.001), and a trend for MK801-induced hyperlocomotion (Fig. 4A). Thus, peripubertal SD increased anxiety in anxiogenic environments and MK-801-induced locomotion in KO mice and enhanced startle reactivity in WT mice (Fig. 4A). By contrast, we found that late adolescent SD only increased exploratory activity in novel environments, irrespectively of genotype (Fig. 4B). These indicated that peripuberty is a more vulnerable period to repeated SD than late adolescence and a genetic vulnerability to oxidative stress modulates the long-lasting effect of peripubertal, but not late adolescence SD.

### Clinical study

#### Effects of early and late PSA on anxiety in EPP

**Fig. 5:**
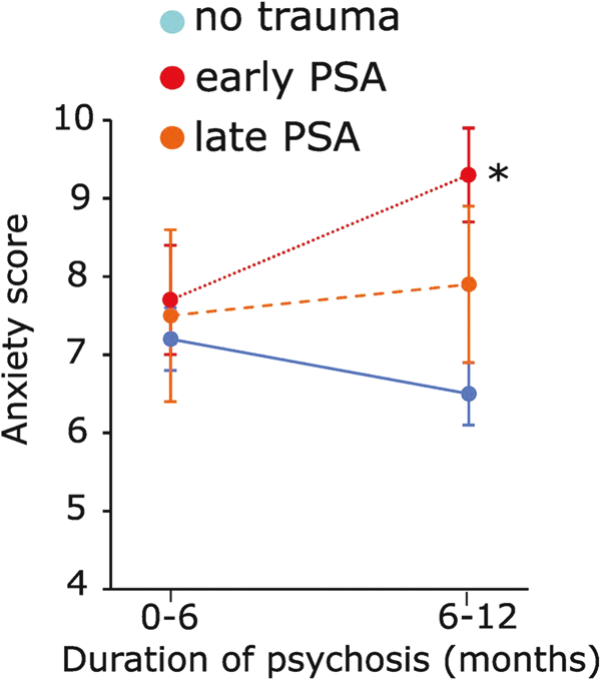
Anxiety score in early psychosis male patients (EPP) with no reported trauma, early physical/sexual abuse (PSA) (first exposure before 12 years old), and late PSA (first exposure between 12 and 16 years old). Anxiety was assessed less than 6 months after psychosis onset and/or 6–12 months after psychosis onset. Data are represented with mean and standard error of measurement. *Indicates significantly different level of anxiety in EPP with early PSA as compared to EPP with no trauma (see text for more on statistical results).

As peripubertal, but not late adolescent SD, increased anxiety-like behaviors in anxiogenic environments in adult male KO mice and increased auditory startle reactivity in adult male WT mice, we explored in a cohort of male EPP whether a “early” first exposure to PSA (before 12 years old) was also associated with higher levels of anxiety. We classified patients (89) into three groups (no trauma/adversity, “early” PSA (before 12), “late” PSA (12–16 years old). The three groups differed for age, but not for socioeconomic status, duration of illness and untreated psychosis (defined as the time between psychosis onset (CAARMS) and admission to the TIPP), and diagnostics (Table 1). When we examined anxiety (as assessed according to [38]), we found trauma effect (*F*_2,80,383_ = 3.471, *P* = 0.036), and trauma × time interaction (*F*_2,53,378_ = 3.652, *P* = 0.033). This was due to a significant increase in anxiety during the second half of the first year of psychosis in the group with early PSA (*F*_1,65,685_ = 4.357, *P* = 0.041) resulting in significantly higher anxiety in this group as compared to the “no trauma” group (*P* < 0.001, Fig. 5). The level of anxiety in EPP with late PSA was intermediate (Fig. 5). These effects on anxiety remained similar when either “age” or “socioeconomical status” was used as covariate, thus supporting the robustness of our conclusions. As secondary outcomes, we also looked at the other psychopathological aspects (positive and negative symptoms, disorganized (cognitive) symptoms, excited features, motor symptoms, depression [38]) and found neither trauma, time, nor trauma × time interaction effects for any of these dimensions (Table S2). During the second half of the first year of psychosis, we observed however a positive correlation between anxiety and positive symptoms, that was especially strong in EPP with early PSA but not significant in EPP with late PSA, probably due to a small sample size (*N* = 7) (*r*_early-trauma_ = 0.756, *P* = 0.001; *r*_late-trauma_ = 0.581, *P* = 0.171; *r*_notrauma_ = 0.385, *P* = 0.005).

**Table 1:** Clinical study: demographic table of male early psychosis patients (EPP).

	No trauma		Early PSA (≤12 years)		Late PSA (12–16 years)		Test value^c^	
*N*^a^	61		21		7		–	
*N* at each time-points^b^ (1st and 2nd clinical assessment period)	36	51	13	16	6	7		
Age, mean (SD), years	24.6 (3.9)		27.1 (4.5)		22.8 (4.1)		*F*(2) = 4.187; *p* = 0.018	
	23.9 (4.1)	24.8 (3.9)	27.7 (5.1)	27.2 (4.0)	23.1 (4.3)	22.9 (4.2)	*F*(2) = 4.028; *p* = 0.024	*F*(2) = 3.500; *p* = 0.035
Socioeconomic status, % (*N*)								
Low	9.8 (6)		23.8 (5)		0 (0)		*λ*(4) = 7.397; *p* = 0.116	
Intermediate	36.1 (22)		52.4 (11)		28.6 (2)			
High	45.9 (28)		23.8 (5)		57.1 (4)			
Duration of illness, mean (SD), years	0.29 (0.10)	0.68 (0.14)	0.35 (0.10)	0.63 (0.09)	0.29 (0.14)	0.66 (0.09)	*F*(2) = 1.293; *p* = 0.283	*F*(2) = 1.128; *p* = 0.330
DUP, mean (SD), days	68.9 (77.6)		50.0 (48.0)		43.0 (28.1)		*F*(2) = 0.882; *p* = 0.417	
Diagnostic, % (*N*)								
Schizophrenia	36.1 (22)		57.1 (12)		42.9 (3)		*λ*(10) = 13.642; *p* = 0.190	
Brief schizophreniform disorder	27.9 (17)		14.3 (3)		14.3 (1)			
Schizoaffective disorder	11.5 (7)		9.5 (2)		14.3 (1)			
Major depressive disorder with psychotic features	0 (0)		9.5 (2)		0 (0)			
Bipolar disorder	11.5 (7)		0 (0)		14.3 (1)			
Others	13.1 (8)		9.5 (2)		14.3 (1)			

*DUP* duration of untreated psychosis, *PSA* physical/sexual abuse.

^a^Number of male early psychosis patients (EPP) included in the clinical study.

^b^Numbers of male EPP in the 1st (<6 months after reaching psychosis threshold) and 2nd (6–12 months after reaching psychosis threshold) clinical assessment time-points. Data specific to each of the two evaluation periods are displayed when relevant.

^c^Test values provided by one way ANOVA (F) or Likelihood ratio (λ) to test for difference between trauma (physical/sexual abuse) groups, at each time point (>6 months and between 6-12 months after psychosis threshold) when relevant.

## Discussion

We used repeated SD in male mice as translational model of PSA to explore how age at the time of exposure to such stress contributes to long-lasting behavioral alterations. We found that SD during peripuberty causes persistent behavioral alterations different from those induced by late adolescent SD. Peripubertal SD increases anxiety-like behavior in anxiogenic environments, potentiates startle response (a defensive reflex to sudden or threatening stimuli) and exacerbates MK-801-induced locomotion, while late adolescent SD causes increased exploration in novel environments. In addition, a novel finding is that redox dysregulation (as in Gclm-KO mice) exerts during peripuberty but not adolescence a permissive influence on SD-induced persistent behavioral alterations, including increased anxiety-like behavior. In line with these, we found that a first exposure to PSA during childhood-peripuberty is also associated with increased anxiety in male EPP.

### The peripubertal period is vulnerable to SD

Numerous studies have investigated the impact of adolescent or adult SD on behavior (mostly in relation to depressive-like behavior [34]), but none has compared the effect of exposure to SD during different periods of postnatal development. The high vulnerability to SD exposure during peripuberty (P30–40), as compared to late adolescence, corroborates findings in rats or mice exposed to chronic unpredictable stress (CUS) [26, 27]. Overall, our results are consistent with other studies on long-lasting behavioral impact of peripubertal SD: increased anxiety [41], no alteration of PPI [42]. The effect of peripubertal SD on social behavior varies however across studies from no impact [41, 42] to reduced social interactions [43, 44]. This discrepancy seems to relate to the mouse strain used to assess social interactions. When the strain differs from the aggressor strain (as in our study), no persisting deficit in social interaction is observed, suggesting that peripubertal SD does not cause generalized persistent social avoidance. Nevertheless, we found reduced social interaction in a subset of mice exposed to peripubertal SD. These susceptible mice do not differentiate however from resilient individuals in other behaviors, including anxiety-like behaviors as also reported in [45]. Although, we did not assess the impact of SD on cognitive functions, others have found that exposure to peripubertal SD impairs extradimensional set-shifting [44, 46] which agrees with the reported association between history of CT and poor cognitive performance [47]. Finally, to the best of our knowledge, MK-801 sensitivity which are affected by peripubertal SD in a genotype-dependent manner has not been previously examined.

Altogether, this highlights that SD occurring at different development periods causes distinct long-lasting behavioral changes and that peripuberty/early adolescence constitutes a time window particularly vulnerable to such stress. Peripubertal SD impacts a broad range of behaviors, affecting cognition, exacerbating MK-801-sensitivity, and favoring anxiety-like phenotype. By contrast, SD at late adolescence/early adulthood produces depressive-like behaviors [21, 48, 49] and only non-persistent anxious phenotype [50]. This supports the notion that the juvenile or early adolescent brain, which undergoes substantial maturation of local brain circuitry and large-scale brain networks, is especially vulnerable to massive surge of stress-related events – such as elevated transient brain activity in circuits mediating emotion, strong catecholamine, and stress hormone release, immune-activation.

### Genetically-induced redox dysregulation alters the effects of peripubertal but not late adolescent SD

Because Gclm-KO mice are prone to oxidative stress and microglia activation [30, 31] and repeated SD disrupts antioxidant systems and activates neuroinflammatory cascades leading to oxidative stress [20–23], we hypothesized that the effects of SD that could be mediated by oxidative stress would be either potentiated or occluded in Gclm-KO mice. Consistent with this, we found that SD affects differently the behavior of KO and WT mice, but only when it occurs during peripuberty and not late adolescence. Thus, peripubertal SD increases anxiety-like behavior and MK-801-sensitivity mostly in Gclm-KO mice, whereas it enhances startle reflex in WT mice only. These suggest that a redox dysregulation during peripuberty strongly interacts with stress to remodel the trajectory of brain maturation, but does not play a significant role in modulating the impact of later stress. During peripuberty/adolescence, prefrontal cortical regions undergo maturation processes and circuit refinement via enhanced plasticity, including increased spine density but also engulfment of dendritic spines by microglia [51, 52]. Oxidative stress perturbs this mechanism at several levels. First, it delays or disrupts maturation of parvalbumin neurons and their enwrapping perineuronal nets that are critical for controlling the opening and closing of plasticity [51, 53]. Second, redox dysregulation/oxidative stress causes activation of microglia [31] and therefore might perturb regulation of synaptic pruning. Altogether, this may contribute to altered connectivity and communication between prefrontal cortex (PFC) and subcortical regions, particularly within circuits undergoing connectivity remodeling during this critical period (e.g. prelimblic cortex – ventral hippocampus – basal lateral amygdala [54]).

### Effect of peripubertal SD and redox dysregulation on MK-801-induced locomotion

NMDA receptor antagonists such as MK-801 induces transient hyperlocomotion that is associated with an increased dopamine release in nucleus accumbens and is blocked by antipsychotics [55]. The low MK-801 sensitivity of non-stressed KO mice is likely due to hypofunction of NMDA receptors [56] due to low brain GSH levels [57]. However, peripubertal but not late adolescent SD unveils a strong MK-801-induced locomotion in adult KO and to lesser extent WT mice. This may be due to a potentiation of dopaminergic signaling in nucleus accumbens. In rats, SD and/or other non-social stress during peripuberty, but not late adolescence, increases the number of active dopaminergic neurons in the ventral tegmental area (VTA) [58] and amphetamine-induced locomotion [27, 59]. CT in humans is also associated with increased amphetamine-induced dopamine release in ventral striatum [60]. A disrupted maturation of PFC by the peripubertal SD might cause this effect as a transient inhibition of PFC during early-life potentiates MK-801-induced locomotion and dopamine release in nucleus accumbens in adults [61]. Thus, an exacerbation of the effects of SD on the PFC maturation, but also on the circuits modulating indirectly the activity of VTA dopaminergic neurons [62–64] might explain the strong enhancement of MK-801-induced locomotion in Gclm-KO mice. Changes in NMDA receptor expression induced by peripubertal SD might also contribute to the enhanced sensitivity to MK-801 following such stress [65].

### Effect of peripubertal SD and redox dysregulation on anxiety

Our study corroborates previous findings that childhood/adolescent stress favors persistent anxiety trait and anxiety disorders [18, 26, 27, 41, 66, 67]. We could hypothesize, given our translational findings in animals, that this could be due partly to oxidative stress-mediated mechanisms as peripubertal SD reduces the drive for exploring anxiogenic environments mostly in adult Gclm-KO mice. A body of evidence shows indeed an association between anxiety and oxidative stress [68–73], expression of enzymes of the GSH-mediating defense system within brain networks implicated in emotion regulation [74], and mitochondrial dysfunction [75] that has been also observed in Gclm-KO mice [76]. Furthermore, adult SD-induced short-term anxiety-like behavior in mice is mitigated by the Nrf2 activator, sulforaphane [21]. However, exposure to SD during peripuberty potentiates also startle response (a defensive reflex related to fear) but only in WT mice. Thus, exploration in anxiogenic environments and startle response, both associated with emotional state, are not concurrent [77] and are affected by peripubertal stress differently in Gclm-KO as compared to WT mice. These two behaviors rely on distinct sub-circuits within a large network of brain regions that regulate anxiety and fear [78, 79]. The decision-making behavior in EPM is guided by a risk assessment of potential threat that involves circuits encompassing the basal amygdala, ventral hippocampus, and mPFC [78–83]. By contrast, the intensity of startle reflex is regulated by more subcortical regions [84]. Consequently, stress and its interaction with redox dysregulation may impact differently these various sub-networks with miscellaneous effects on anxiety (such as generalized anxiety) and fear-related behaviors (such as observed in phobia or post-traumatic stress disorder).

### Relationship between time of first exposure to PSA and anxiety and in early psychosis patients

Human studies report high levels of anxiety in adults exposed to childhood PSA [85], but to our knowledge the relationship between age at first exposure to such trauma and anxiety has not been previously explored. This question is relevant since persistent high anxiety through childhood and adolescence is a risk for psychosis [86], and the association between history of abuse and psychotic symptoms is partly mediated by anxiety [87, 88]. In the present study, male EPP with a first exposure to PSA before 12 display higher anxiety than non-traumatized EPP. This difference manifests 6–12 months after psychosis onset. Although it is unclear why no group difference was observed during the first months following psychosis onset, these data suggest that male EPP reporting a first PSA before 12 years old are prone to anxiety. We found however no effect of PSA on any of the other psychopathological dimensions, despite a weak association between positive symptoms and physical or sexual abuse revealed by a meta-analysis in people with psychosis [8]. Explanation for this apparent discrepancy may include the relatively small size of our cohort, but also the fact that the meta-analysis considered both sexes and PSA until age of 18 years regardless of the time of exposure. Nevertheless in our study, anxiety correlates strongly with positive symptoms, particularly in EPP exposed to early PSA. Thus, this provides additional evidence for associations between PSA, anxiety and positive symptoms. Together with previous report that EPP subjected to PSA before 12 present worse functional outcome than those exposed later to these trauma [5], this supports that early-life (childhood-peripuberty) as a particularly vulnerable period to PSA. This is in line with our preclinical study although the time windows of abuse exposure in the clinical study do not fully coincide with those of the preclinical study. Thus based on sexual, behavioral and brain maturation, the period considered for early PSA (from birth to 11 years old) corresponds to childhood-to-early peripuberty, while the time window defined for late PSA (from 12 to 16 years old) relates to mid-to-late peripuberty [89, 90]. The late adolescence in humans is typically considered to extend from about 16 to 21 years old. The present study has additional limitations to be addressed in future investigations. This includes larger sample sizes with inclusion of women as a separate cohort due to gender-specific stress effects [91–93] (also in rats, females are more resistant than males to peripubertal stress [94]), assessment of anxiety with more specific tools, more complete information regarding antipsychotic and anxiolytic (often used pro re nata) medication, so this can be used as covariate in the analyses. Finally, individuals subjected to PSA during childhood can be exposed to recurrent abuses and others types of trauma/adversities throughout adolescence. Therefore, our data on the effect of first exposure to abuse before age of 12 could be partly driven by the cumulative exposure to multiple traumatic events. However in our cohort, about 75% EPP exposed to PSA before 12 years old did not report any additional traumatic events. Finally, given the results of our mouse study, an investigation on the mediating role of redox dysregulation to abuse-induced persistent impact is warranted in humans, irrespective of the patient diagnosis.

To conclude, childhood/early adolescence is a vulnerability period to PSA exposure in both male mice and EPP. These findings support the need to pay special attention to individuals with a history of early abuse and develop tailored intervention that could include therapeutic approach targeting oxidative stress.

## Supplementary information

Supplemental material
