# Supplementary material for: Time of exposure to social defeat stress during childhood and adolescence and redox dysregulation on long-lasting behavioral changes, a translational study
Source: Transl Psychiatry. 2022 Sep 26;12:413. doi: 10.1038/s41398-022-02183-7 (PMC9512907; doi:10.1038/s41398-022-02183-7)
Supplement: Supplementary file 1 — Supplemental material [file 41398_2022_2183_MOESM1_ESM.docx]

**Supplemental material**

Schnider M. et al. : “Time of exposure to social defeat stress during childhood and adolescence and redox dysregulation on long-lasting behavioral changes, a translational study. ”

**MATERIAL AND METHODS**

**Preclinical study**

SD protocol. Experimental mice were exposed to a repeated SD stress (adapted from (1)) either during peripuberty (from P30 to P40) or late adolescence (from P50 to P60). The stress consisted in introducing an experimental mouse into the cage (type IIL) of a single-housed adult male SJL mouse (from Charles River, France). The young experimental mouse was typically attacked by the adult SJL within the first minutes and rapidly adopted a subordinate behavior. If no attack occurred during the first three minutes, the experimental mouse was placed into the cage of another single-caged SJL mouse. After 10 minutes of physical interactions or as soon as 10 attacks from the SJL mouse occurred, a perforated Plexiglas partition was introduced to separate the two mice until the next day. To avoid behavioral habituation, the socially defeated animal was moved the following day into the cage of another SJL mouse, and the same protocol of SD was applied. This was repeated for eleven consecutive days and the experimental mice were then single-housed until behavioral testing (Fig. 1). Mice were kept isolated following the period of stress exposure as social grouping dampens down the long-term stress-induced behavioral alterations. Control non-stressed mice were kept grouped and gently handled during the same period of stress exposure (either P30-40 or P50-60). The choice of attribution of each animal to a stress or non-stress protocol was done arbitrarily but took into account that mice originated from the same litter should be distributed into stressed and non-stressed groups.

Behavioral tests

The experimenter was not aware of the mice genotype and/or treatment during the behavioral experiments.

Elevated-plus-maze. Anxiety-like behavior was measured on an elevated-plus-maze (arms: 30 x 5 cm). The light intensity was set to approximately 30-lux on the open arms. Mice were placed in the central zone of the maze facing one of the close arms and allowed to freely explore the maze for 5 minutes. The locomotor activity on the open and close arms was tracked using the Any-maze tracking system (Stoelting Co., Ireland).

Open field. Locomotor activity was evaluated in white open field boxes (40 x 40 x 40 cm, length x wide x height). The room´s light intensity was set to 100 lux in the central zone. Mice were placed in the center and allowed to freely explore the arena for 60 minutes. The total distance moved within the arena and the time spent in its centre zone (13 x 13 cm) were recorded.

Social interaction. Social behavior was tested in a T-maze consisting of a start arm and two side-arms. A box was placed at each end of the side arms and separated from the maze by a metal grid (1.2 cm space). The test was performed under dimmed light conditions (~30 lux). During the habituation phase, which lasted 10 min for two consecutive days, mice were allowed to explore freely the maze containing an empty box at each side-arm end. On the third day, a social interaction test was performed for 5 minutes. An adult unfamiliar C57Bl/6J male mouse was placed in a box at the end of one arm while the box at the end of the other arm contained a red egg-shaped object. The placing of the mouse and object was randomly assigned. The locomotor activity of mice was tracked with Anymaze software. The cumulative time during which the animal´s head was in the interaction zone of each box (within 2 cm from the box) and the total distance moved were measured.

Pre-pulse inhibition. The pre-pulse inhibition (PPI) of an acoustic stimulus was measured in startle chambers for mice (Med Associates, USA). This test assesses sensory-motor gating that is affected in schizophrenia (2). Mice were exposed to a series of discrete trials including pulse-alone trials, pre-pulse alone trials, pre-pulse followed by pulse trials, and no-stimulus trials. The pulse (40 ms) and pre-pulse (20 ms) stimuli were white noise played at two intensities for the pulse (100, 110 dB) and three intensities for the pre-pulse (71, 77, 83 dB). The stimulus delay between pulse and pre-pulse was set to 100 ms. A background noise was set to 65 dB. The inter-trial interval ranged between 10-15 seconds. Mice were placed into a small Plexiglas box inside the startle chamber and body movements upon the sound presentation were recorded. The signal amplitude of the body movement during the first 66 ms after the stimulus onset was used as startle response. A session began after a 2-minutes habituation period and consisted of 3 phases. During the first phase, the mice were exposed to both pulse stimuli to habituate and stabilize the animal´s response to the acoustic stimuli. During the second phase, the pre-pulse inhibition was measured. The animals were exposed to 10 blocks of discrete test trials. Each block consisted of: 2 “pulse alone” trials, 3 “pre-pulse alone” trials, 6 “pre-pulse followed by pulse” trials (all combinations of pre-pulse and pulse intensities) and 1 “no-stimulus” trial. The last phase consisted again of 6 “pulse alone” trials identical to the first block. The percentage of pre-pulse inhibition was calculated for each pulse intensity as follows: 100% x (1–[mean response on “pre-pulse followed by pulse” trials/mean response on “pulse alone” trials]). In addition, the startle response to pulses (in absence of pre-pulse) was analyzed.

MK-801 sensitivity. Low doses of NMDA receptor antagonists produce transient symptoms and cognitive deficits resembling those observed in schizophrenia patients. In rodents, injection of a low doses of NMDA receptor antagonists causes a transient hyperlocomotion. Here, we measured the locomotor response to the non-competitive N-methyl-d-aspartate (NMDA) receptor antagonist dizocilpine (MK-801) in a white open field box (40 cm x 40 cm x 40 cm, length x wide x height). Mice were first habituated to the arena for 20-minutes. Next, animals received an i.p. injection of sterile saline solution (0.9% NaCl) and the locomotor activity was recorded for another 20-minutes. Finally, mice were injected with MK-801 (Sigma) (0.2 mg/kg, i.p, volume 5 ml/kg) and the locomotor activity recorded during 90-minutes. The distance moved was measured in 5-minute-bins.

**Clinical study**

Subjects. Early psychotic patients (EPP) were enrolled within a Treatment and Early Intervention in Psychosis Program (TIPP, Lausanne University Hospital) (3). Inclusion criteria comprised age between 18 and 35, having crossed the psychosis threshold according to the Comprehensive Assessment of At-Risk Mental States (CAARMS) criteria (4), no previous treatment with antipsychotic medication for more than 24 weeks, no psychosis related to intoxication or organic brain disease, intelligence quotient ≥ 70, and ability to provide informed consent. Each EPP was closely followed up by a case manager and a psychiatrist. A confirmed diagnostic for each EPP was obtained at latest by the end of the TIPP program (3 years). Initially 482 EPP were considered from which 15 were excluded for clinical reasons, 189 because of absence of information about physical/sexual abuses or age at first abuse exposure, and 148 due to lack of available clinical assessment during the first year of psychosis. From the remaining 130 EPP with complete clinical data and trauma information, only men (89 individuals, see Table 1) were investigated to better translate the results from our preclinical study on male mice. Furthermore, the number of women with complete clinical and trauma data was too small to perform a complementary analysis on this population. The local Ethics Committee (*Commission Cantonale d’Ethique de la Recherche sur l’Etre Humain*) granted access to TIPP clinical data including demographic, PANSS and trauma history.

Statistics. Mixed effects models repeated measure analysis of variance (MMRM) were used to analyze differences in psychopathological measurements among the three trauma groups (no trauma, early, late) over the first year of psychosis (0-6 and 6-12 months). Anxiety and the other psychopathological dimensions were considered in separate MMRM models, where the optimal within subject covariance matrix was systematically selected (AIC coefficient tested through *unstructured, autoregressive, compound symmetric* and *Toeplitz* structures, including heterogeneous versions to overcome the lack of homogeneity of variances). We were interested in main, simple and interaction effects, but also in multiple comparisons (post hoc) when a signal was present. These statistical analyses were performed with SPSS (IBM, USA). Antipsychotic and anxiolytic medication were not used as covariates due to missing information for a significant proportion of patients (for antipsychotics: 27 out of 55 and 39 out of 74 patients respectively at the first and second time point of the clinical assessment; for anxiolytics: 26 out of 55 and 39 out of 74 patients respectively at the first and second time point of the clinical evaluation.

**RESULTS**

**Preclinical study**

**Table S1**. Timings for the repeated social defeat stress (SD) and the subsequent behavioral assessments with the number (#) of mice in each experimental group. S+ and S- mean respectively stressed and non-stressed (control) mice.

| Experiment | Social defeat (SD) | # Gclm-WT  (S+ / S-) | # Gclm-KO  (S+ / S-) | approximate age at testing |
| --- | --- | --- | --- | --- |
| Elevated-plus-maze | *PND30-40*  *PND50-60* | 13 / 10  10 / 10 | 12 / 10  1 0/ 10 | PND70  PND90 |
| Open field | *PND30-40*  *PND50-60* | 13 / 10  10 / 10 | 12 / 10  10 / 10 | PND75  PND95 |
| Social interaction test | *PND30-40*  *PND50-60* | 12 / 10  10 / 10 | 12 / 10  10 / 10 | PND80  PND100 |
| Pre-pulse inhibition  Startle response | *PND30-40*  *PND50-60* | 10 / 10  10 / 10 | 10 / 10  10 / 10 | PND90  PND110 |
| Sensitivity to MK-801 | *PND30-40*  *PND50-60* | 13 / 10  10 / 10 | 12 / 9  10 / 10 | PND135  PND155 |


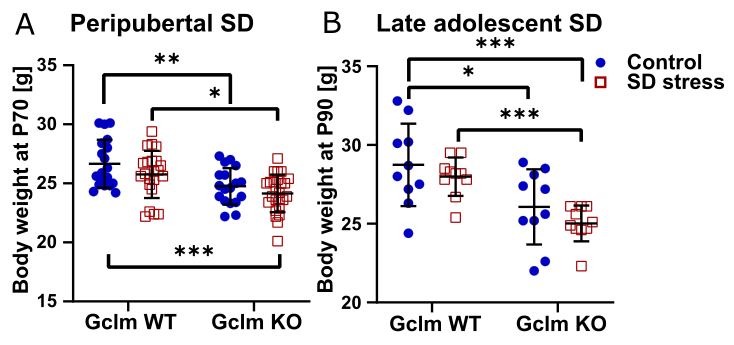


**Figure S1.** Body weight of WT and Gclm-KO mice measured 30 days after the end of the peripubertal and the late adolescent social defeat (SD) stress, respectively. Significant effect of genotype, but no SD effect in both (A) and (B). Control mice are non-stressed individuals. Note that data in (A) also include mice that were not tested in the behavioral paradigms of the present study. Data are presented with the mean and s.d. Horizontal line for each group depicts the mean. * p<0.05, ** p<0.01, *** p<0.001.


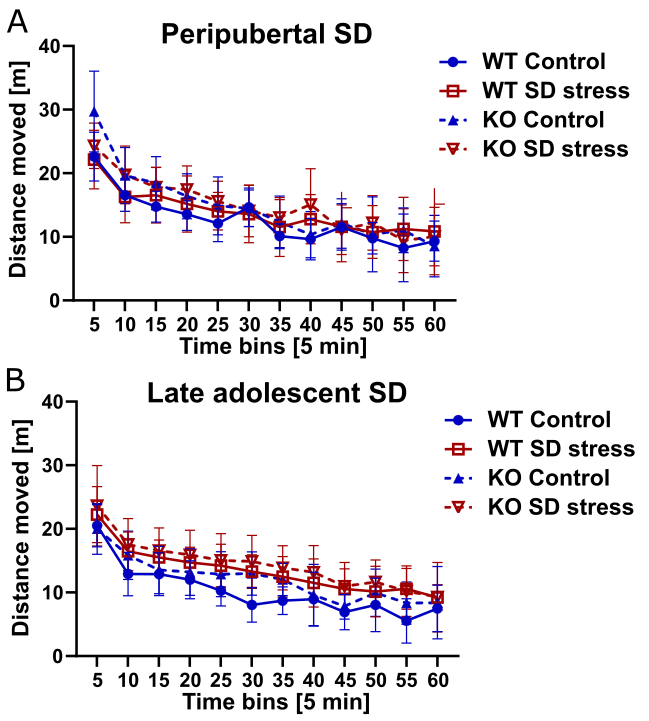


**Figure S2.** Locomotor activity (throughout a 60-min period in open field) of non-stressed WT and Gclm-KO mice and WT and Gclm-KO mice exposed to either peripubertal (A) or late adolescent (B) social defeat (SD) stress. Control mice are non-stressed individuals. Data are represented with the mean and s.d.


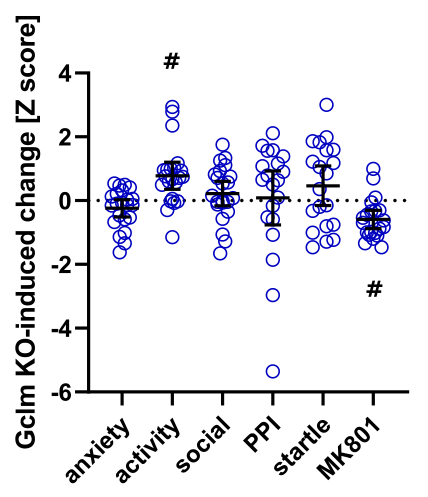


**Figure S3.** Genotype-induced changes of anxiety-like behavior in anxienic environments, exploratory activity in novel environments, social interaction, pre-pulse inhibition (PPI), acoustic startle reflex, and MK-801-induced locomotor activity. Behavior of non-stressed Gclm-KO are compared to non-stressed WT mice (non-stressed individuals from the peripubertal and late adolescent cohorts pooled together). Data are presented with the mean and 95% confidence interval. # indicates that non-stressed Gclm-KO mice behaved differently to WT mice in exploratory activity and MK-801-induced locomotion (p<0.05, sign test).

**Clinical study**

**Table S2.** PANSS assessments of male early psychotic patients (EPP) respectively 0-6 and 6-12 months following psychosis onset. EPP are classified in 3 groups: EPP reporting no traumatic/adverse events (no), EPP with a fist exposure to physical/sexual abuse (PSA) before 12 years old (early), and EPP with a fist exposure to PSA between 12 and 16 years old (late). The seven-factor model proposed by Emsley et al. (5) was chosen to categorize the PANSS items into 7 psychopathological dimensions: anxiety, Positive symptoms, Negative symptoms, disorganized (cognitive) symptoms, excited features, motor symptoms, and depression. Numbers in parenthesis are the numbers of EPP in each group. The only significant trauma (PSA) effect was for anxiety (Mixed effects models repeated measure analysis of variance (MMRM), see details in main text). S.e.m.: standard error of measurement.

| Duration of psychosis |  | Anxiety | | Positive | | Negative | | Disorganized | | Excited | | Motor | | Depression | |
| --- | --- | --- | --- | --- | --- | --- | --- | --- | --- | --- | --- | --- | --- | --- | --- |
|  | Trauma | Mean | s.e.m. | Mean | s.e.m. | Mean | s.e.m. | Mean | s.e.m. | Mean | s.e.m. | Mean | s.e.m. | Mean | s.e.m. |
| 0-6  months | No  (*36*) | 7.2 | 0.4 | 14.2 | 0.8 | 16.5 | 1.1 | 9.9 | 0.6 | 5.5 | 0.4 | 3.6 | 0.3 | 5.3 | 0.3 |
|  | Early PSA (*13*) | 7.7 | 0.7 | 13.8 | 1.4 | 17.8 | 1.9 | 8.6 | 1.0 | 6.3 | 0.7 | 3.9 | 0.4 | 5.8 | 0.6 |
|  | Late PSA (*6*) | 7.5 | 1.1 | 15.9 | 2.1 | 20.7 | 2.8 | 8.6 | 1.6 | 6.9 | 1.0 | 4.0 | 0.7 | 5.6 | 0.9 |
| 6-12 months | No  (*51*) | 6.5 | 0.4 | 12.1 | 0.6 | 17.0 | 1.0 | 8.8 | 0.4 | 5.4 | 0.3 | 3.6 | 0.2 | 5.1 | 0.3 |
|  | Early PSA (*16*) | 9.3 | 0.6 | 14.4 | 1.0 | 18.8 | 1.7 | 8.3 | 0.8 | 6.7 | 0.6 | 4.0 | 0.4 | 5.9 | 0.5 |
|  | LatePSA (*7*) | 7.9 | 1.0 | 14.6 | 1.6 | 18.9 | 2.7 | 9.6 | 1.2 | 6.0 | 0.9 | 3.7 | 0.6 | 5.3 | 0.8 |

**References**

1. Golden SA, Covington HE, 3rd, Berton O, Russo SJ. A standardized protocol for repeated social defeat stress in mice. Nature protocols. 2011;6:1183-91.

2. Swerdlow NR, Light GA, Thomas ML, Sprock J, Calkins ME, Green MF, et al. Deficient prepulse inhibition in schizophrenia in a multi-site cohort: Internal replication and extension. Schizophrenia research. 2018;198:6-15.

3. Baumann PS, Crespi S, Marion-Veyron R, Solida A, Thonney J, Favrod J, et al. Treatment and early intervention in psychosis program (TIPP-Lausanne): Implementation of an early intervention programme for psychosis in Switzerland. Early intervention in psychiatry. 2013;7:322-8.

4. Yung AR, Yuen HP, McGorry PD, Phillips LJ, Kelly D, Dell'Olio M, et al. Mapping the onset of psychosis: the Comprehensive Assessment of At-Risk Mental States. The Australian and New Zealand journal of psychiatry. 2005;39:964-71.

5. Emsley R, Rabinowitz J, Torreman M. The factor structure for the Positive and Negative Syndrome Scale (PANSS) in recent-onset psychosis. Schizophrenia research. 2003;61:47-57.
